# Supplementary material for: Peak Slope Ratio of the Recruitment Curves Compared to Muscle Evoked Potentials to Optimize Standing Configurations with Percutaneous Epidural Stimulation after Spinal Cord Injury
Source: J Clin Med. 2024 Feb 27;13(5):1344. doi: 10.3390/jcm13051344 (PMC10932170; doi:10.3390/jcm13051344)
Supplement: Supplementary file 1 [file jcm-13-01344-s001.zip › jcm-2844750-supplementary.pdf]

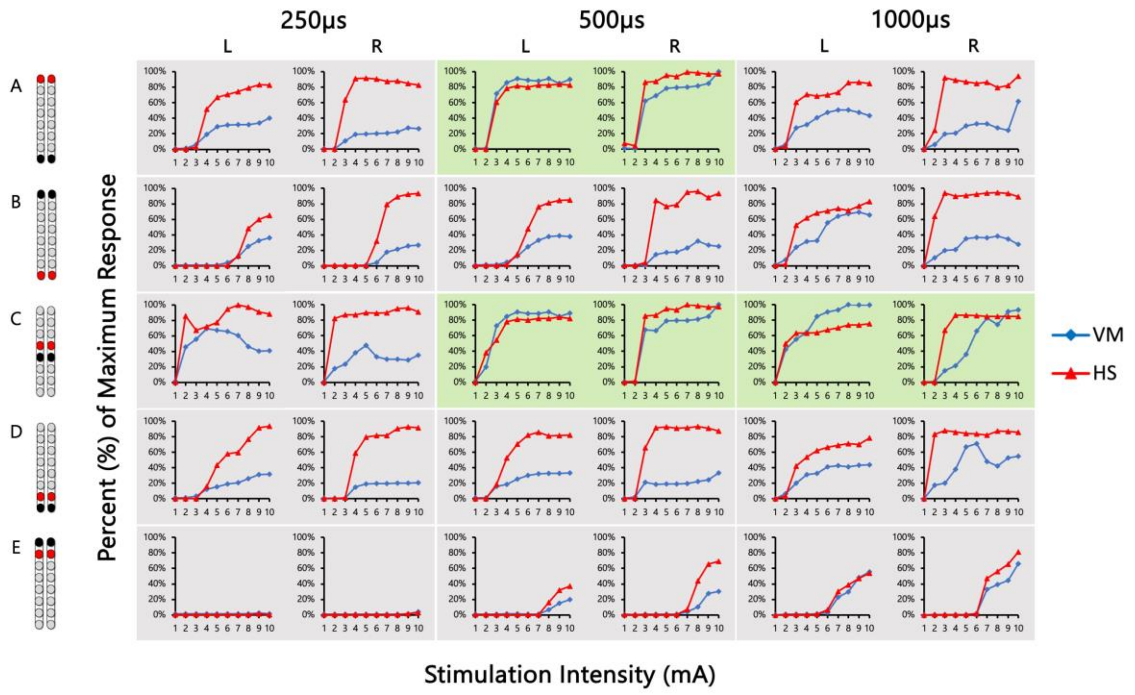

Figure S1. Supine SCES-evoked recruitment curves of the knee extensor and flexor muscles for 0881. Supine SCES-evoked recruitment curves of the left and right VM and HS muscles collected by stimulating at 2Hz from (1-10mA) using the 5 standardized SCES configurations each collected at pulse durations of 250, 500, and 1000µs. (A) Wide-field configuration with caudal cathodes and rostral anodes. (B) Wide-field configuration with rostral cathodes and caudal anodes. (C) Central narrow-field configuration with rostral anodes and caudal cathodes. (D) Caudal narrow-field configuration with rostral anodes and caudal cathodes. (E) Rostral narrow-field configuration with rostral cathodes and caudal anodes. Recruitment curves highlighted green indicate an overall higher extensor to flexor muscle response, while curves highlighted gray represent an overall higher flexor to extensor muscle response. Responses were normalized to the maximum response of each muscle across all amplitudes in all configurations. Cathode and anode electrodes are shown in black and red, respectively. VM: vastus medialis; HS: hamstrings; mA: milliampere; µs: microsecond; L: left; R: right.

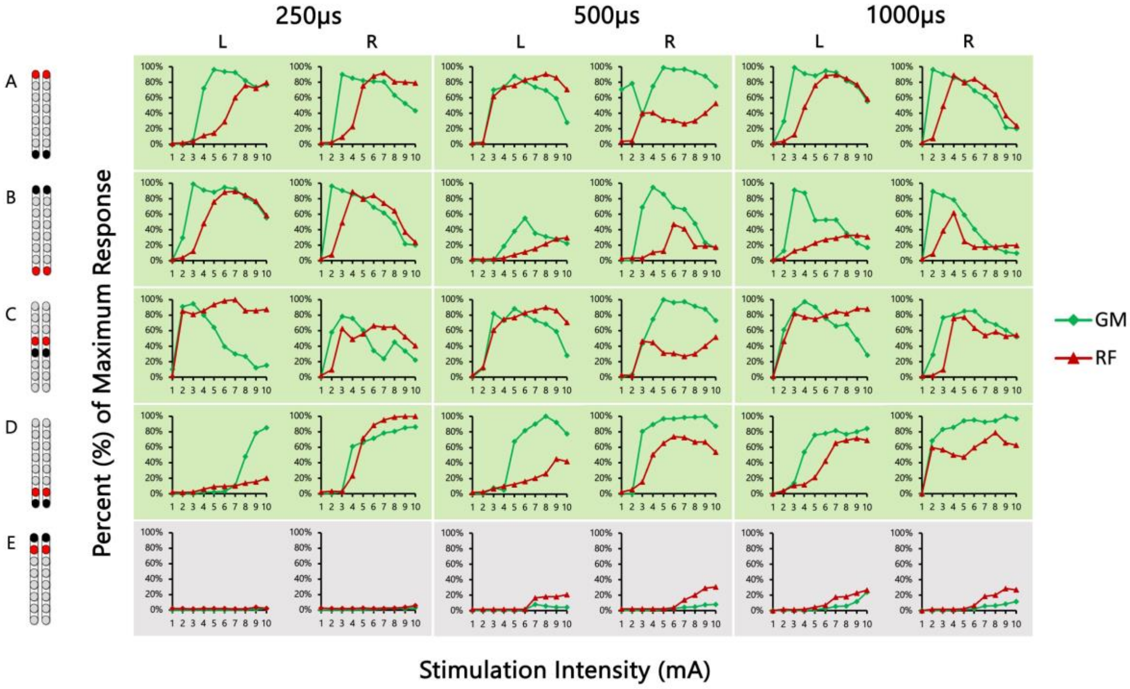

Figure S2. Supine SCES-evoked recruitment curves of the hip extensor and flexor muscles for 0881. Supine SCES-evoked recruitment curves of the left and right GM and RF muscles collected by stimulating at 2Hz from (1-10mA) using the 5 standardized SCES configurations each collected at pulse durations of 250, 500, and 1000µs. (A) Wide-field configuration with caudal cathodes and rostral anodes. (B) Wide-field configuration with rostral cathodes and caudal anodes. (C) Central narrow-field configuration with rostral anodes and caudal cathodes. (D) Caudal narrow-field configuration with rostral anodes and caudal cathodes. (E) Rostral narrow-field configuration with rostral cathodes and caudal anodes. Recruitment curves highlighted green indicate an overall higher extensor to flexor muscle response, while curves highlighted gray represent an overall higher flexor to extensor muscle response. Responses were normalized to the maximum response of each muscle across all amplitudes in all configurations. Cathode and anode electrodes are shown in black and red, respectively. GM: gluteus medius; RF: rectus femoris; mA: milliampere; µs: microsecond; L: left; R: right.

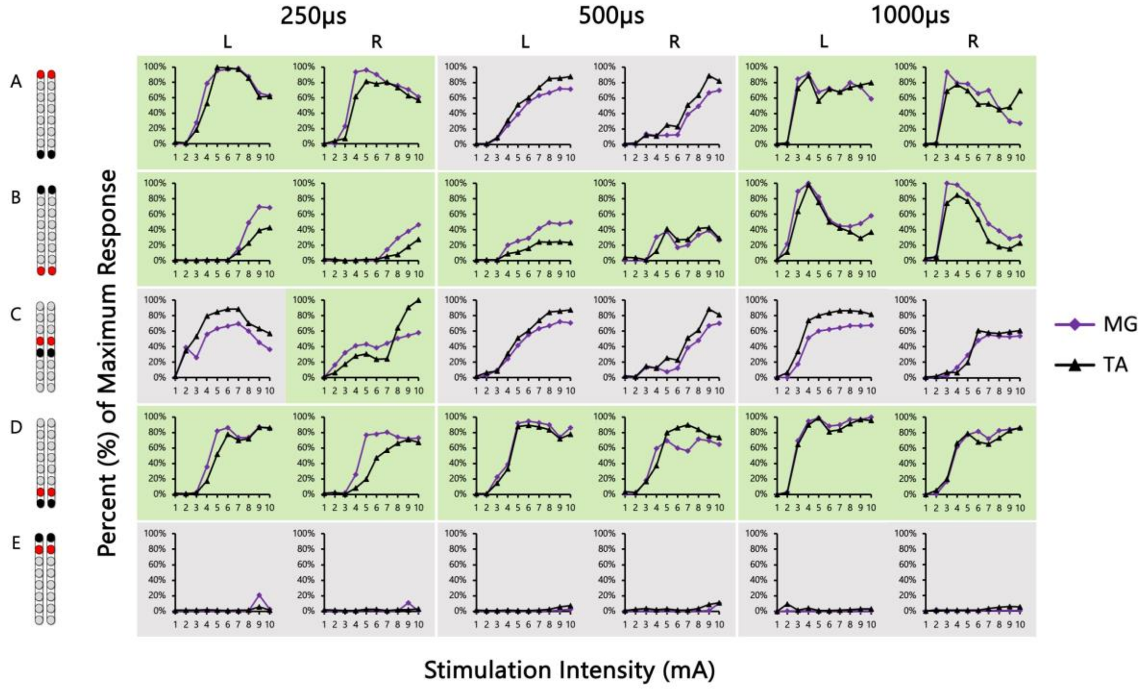

Figure S3. Supine SCES-evoked recruitment curves of the ankle extensor and flexor muscles for 0881. Supine SCES-evoked recruitment curves of the left and right MG and TA muscles collected by stimulating at 2Hz from (1-10mA) using the 5 standardized SCES configurations each collected at pulse durations of 250, 500, and 1000μs. (A) Wide-field configuration with caudal cathodes and rostral anodes. (B) Wide-field configuration with rostral cathodes and caudal anodes. (C) Central narrow-field configuration with rostral anodes and caudal cathodes. (D) Caudal narrow-field configuration with rostral anodes and caudal cathodes. (E) Rostral narrow-field configuration with rostral cathodes and caudal anodes. Recruitment curves highlighted green indicate an overall higher extensor to flexor muscle response, while curves highlighted gray represent an overall higher flexor to extensor muscle response. Responses were normalized to the maximum response of each muscle across all amplitudes in all configurations. Cathode and anode electrodes are shown in black and red, respectively. MG: medial gastrocnemius; TA: tibialis anterior; mA: milliampere; μs: microsecond; L: left; R: right.

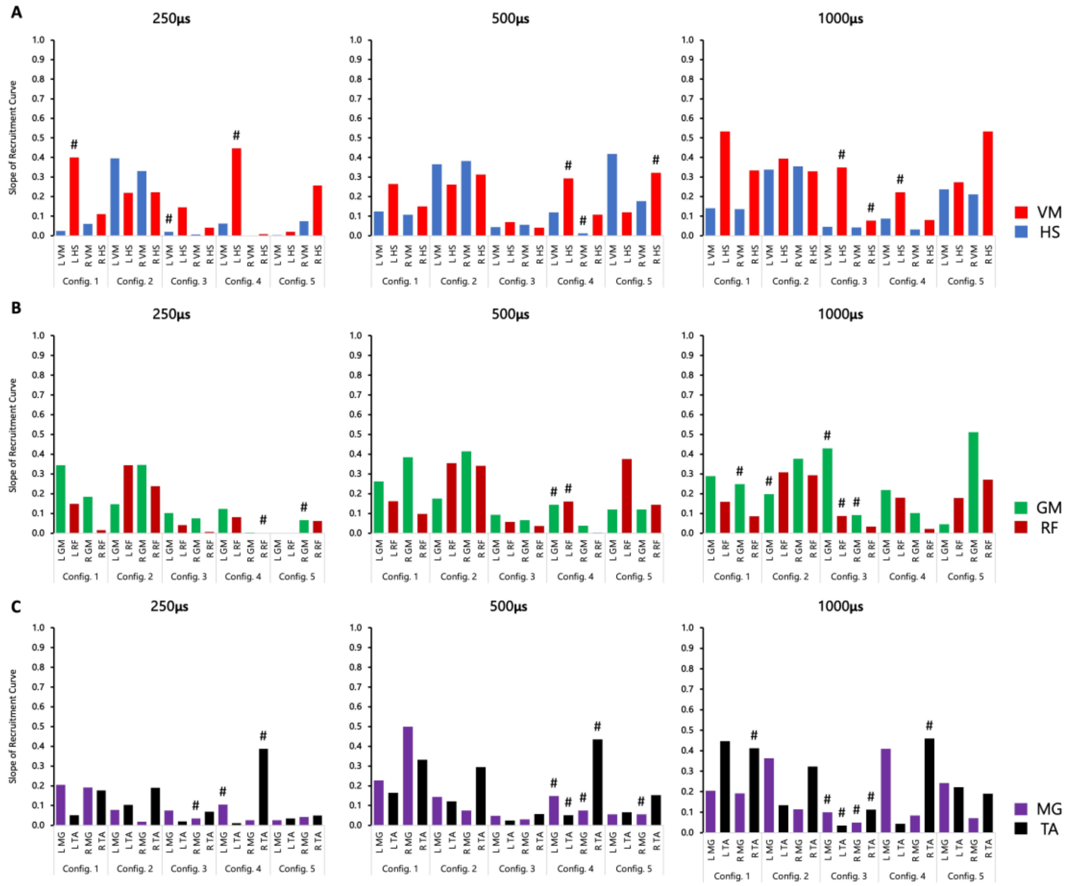

Figure S4. Individual peak slope values of key extensor and flexor muscle pairs for 0881. Maximal peak slope of the SCES-evoked recruitment curves was determined for the knee, hip, and ankle extensors and flexors using the five standardized configurations. (A) Peak slope for the VM and HS at 250, 500, and 1000µs. (B) Peak slope for the GM & RF at 250, 500, and 1000µs. (C) Peak slope for the GM & RF at 250, 500, and 1000µs. Config. 1: wide-field configuration with caudal cathodes and rostral anodes; Config. 2: wide-field configuration with rostral cathodes and caudal anodes; Config. 3: central narrow-field configuration with rostral anodes and caudal cathodes; Config. 4: caudal narrow-field configuration with rostral anodes and caudal cathodes; Config. 5: rostral narrow-field configuration with rostral cathodes and caudal anodes. VM: vastus medialis; HS: hamstrings; GM: gluteus medius; RF: rectus femoris; MG: medial gastrocnemius; TA: tibialis anterior. # Indicates sigmoidal fit had an R2 < 0.8.

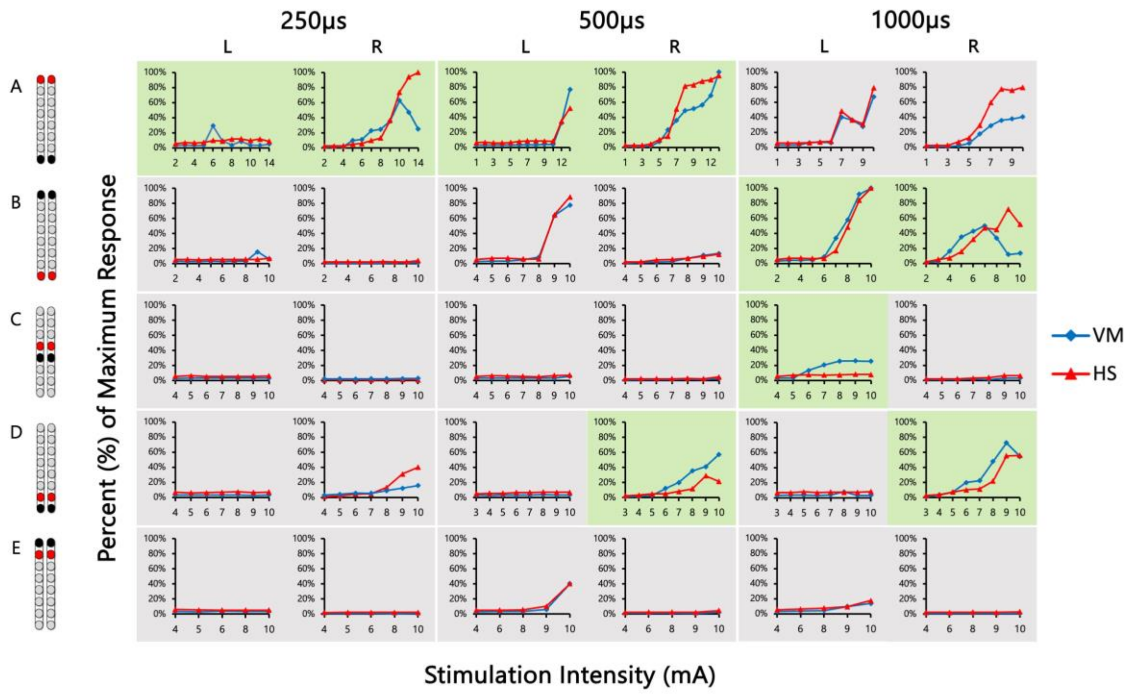

Figure S5. Supine SCES-evoked recruitment curves of the knee extensor and flexor muscles for 0883. Supine SCES-evoked recruitment curves of the left and right VM and HS muscles collected by stimulating at 2Hz from (1-10mA) using the 5 standardized SCES configurations each collected at pulse durations of 250, 500, and 1000μs. (A) Wide-field configuration with caudal cathodes and rostral anodes. (B) Wide-field configuration with rostral cathodes and caudal anodes. (C) Central narrow-field configuration with rostral anodes and caudal cathodes. (D) Caudal narrow-field configuration with rostral anodes and caudal cathodes. (E) Rostral narrow-field configuration with rostral cathodes and caudal anodes. Recruitment curves highlighted green indicate an overall higher extensor to flexor muscle response, while curves highlighted gray represent an overall higher flexor to extensor muscle response. Responses were normalized to the maximum response of each muscle across all amplitudes in all configurations. Cathode and anode electrodes are shown in black and red, respectively. VM: vastus medialis; HS: hamstrings; mA: milliampere; μs: microsecond; L: left; R: right.

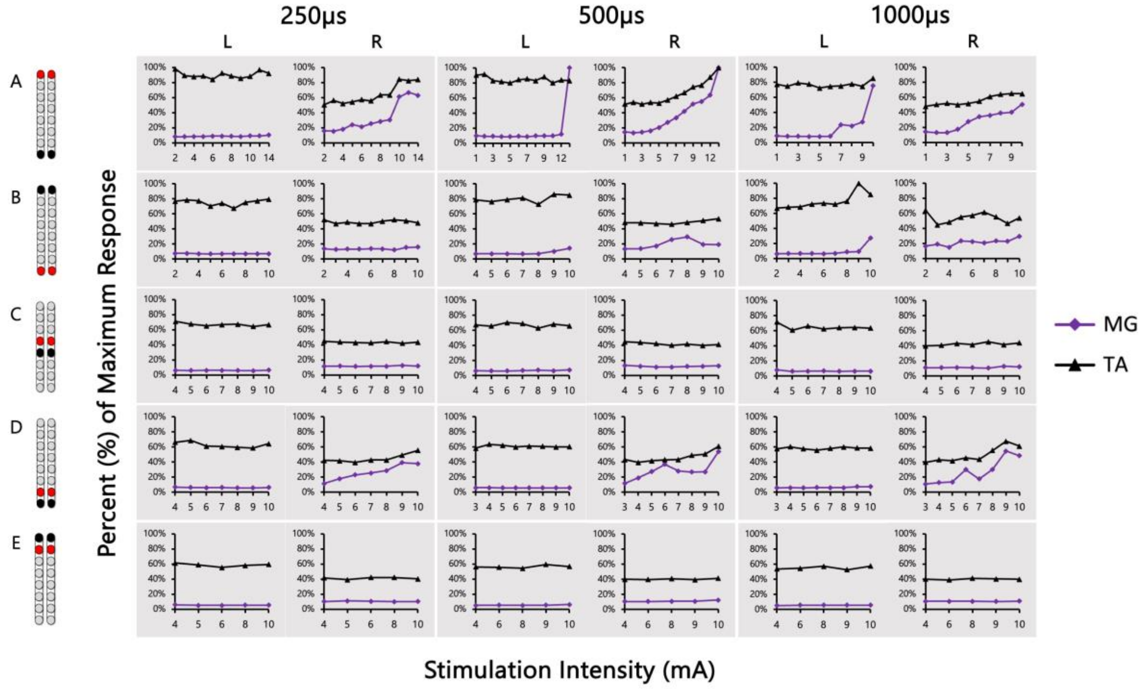

Figure S6. Supine SCES-evoked recruitment curves of the ankle extensor and flexor muscles for 0883. Supine SCES-evoked recruitment curves of the left and right MG and TA muscles collected by stimulating at 2Hz from (1-10mA) using the 5 standardized SCES configurations each collected at pulse durations of 250, 500, and 1000μs. (A) Wide-field configuration with caudal cathodes and rostral anodes. (B) Wide-field configuration with rostral cathodes and caudal anodes. (C) Central narrow-field configuration with rostral anodes and caudal cathodes. (D) Caudal narrow-field configuration with rostral anodes and caudal cathodes. (E) Rostral narrow-field configuration with rostral cathodes and caudal anodes. Recruitment curves highlighted green indicate an overall higher extensor to flexor muscle response, while curves highlighted gray represent an overall higher flexor to extensor muscle response. Responses were normalized to the maximum response of each muscle across all amplitudes in all configurations. Cathode and anode electrodes are shown in black and red, respectively. MG: medial gastrocnemius; TA: tibialis anterior; mA: milliampere; μs: microsecond; L: left; R: right.

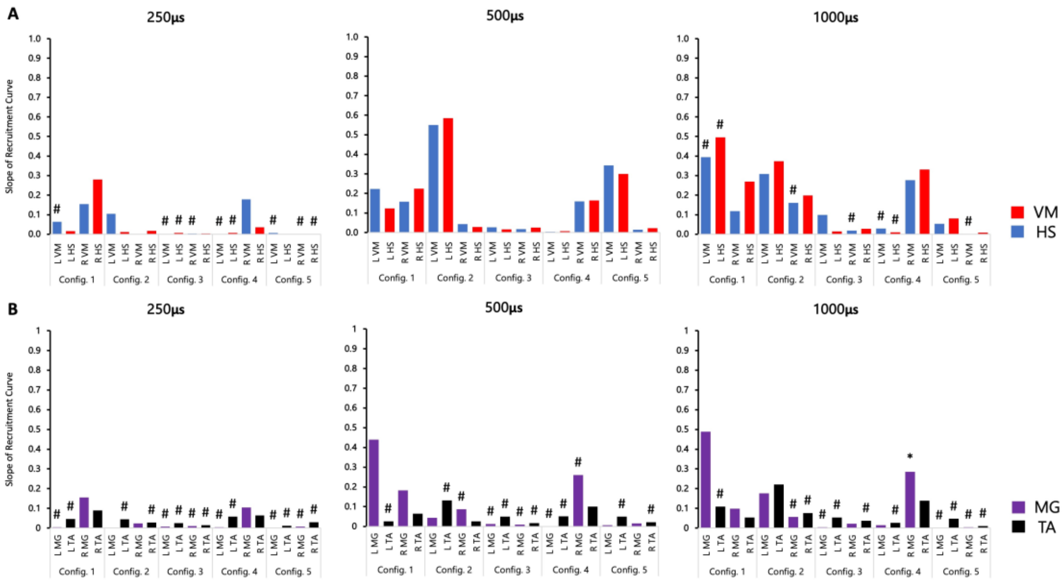

Figure S7. Individual peak slope values of key extensor and flexor muscle pairs for 0883. Maximal peak slope of the SCES-evoked recruitment curves was determined for the knee, hip, and ankle extensors and flexors using the five standardized configurations. (A) Peak slope for the VM and HS at 250, 500, and 1000µs. (B) Peak slope for the GM & RF at 250, 500, and 1000µs. (C) Peak slope for the GM & RF at 250, 500, and 1000µs. Config. 1: wide-field configuration with caudal cathodes and rostral anodes; Config. 2: wide-field configuration with rostral cathodes and caudal anodes; Config. 3: central narrow-field configuration with rostral anodes and caudal cathodes; Config. 4: caudal narrow-field configuration with rostral anodes and caudal cathodes; Config. 5: rostral narrow-field configuration with rostral cathodes and caudal anodes. VM: vastus medialis; HS: hamstrings; MG: medial gastrocnemius; TA: tibialis anterior. # Indicates sigmoidal fit had an $R2 < 0.8$.

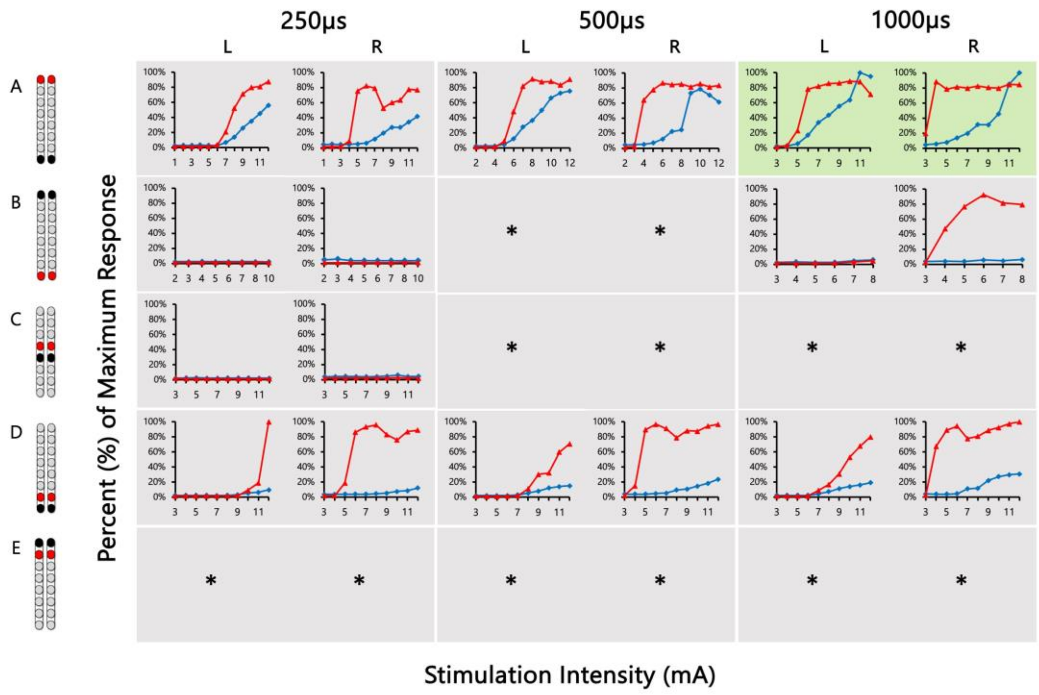

Figure S8. Supine SCES-evoked recruitment curves of the knee extensor and flexor muscles for 0884. Supine SCES-evoked recruitment curves of the left and right VM and HS muscles collected by stimulating at 2Hz from (1-10mA) using the 5 standardized SCES configurations each collected at pulse durations of 250, 500, and 1000μs. (A) Wide-field configuration with caudal cathodes and rostral anodes. (B) Wide-field configuration with rostral cathodes and caudal anodes. (C) Central narrow-field configuration with rostral anodes and caudal cathodes. (D) Caudal narrow-field configuration with rostral anodes and caudal cathodes. (E) Rostral narrow-field configuration with rostral cathodes and caudal anodes. Recruitment curves highlighted green indicate an overall higher extensor to flexor muscle response, while curves highlighted gray represent an overall higher flexor to extensor muscle response. Responses were normalized to the maximum response of each muscle across all amplitudes in all configurations. Cathode and anode electrodes are shown in black and red, respectively. VM: vastus medialis; HS: hamstrings; mA: milliampere; μs: microsecond; L: left; R: right. *Indicates not enough EMG data was available to construct SCES-evoked recruitment curve and/or stimulation resulted in increased abdominal activity.

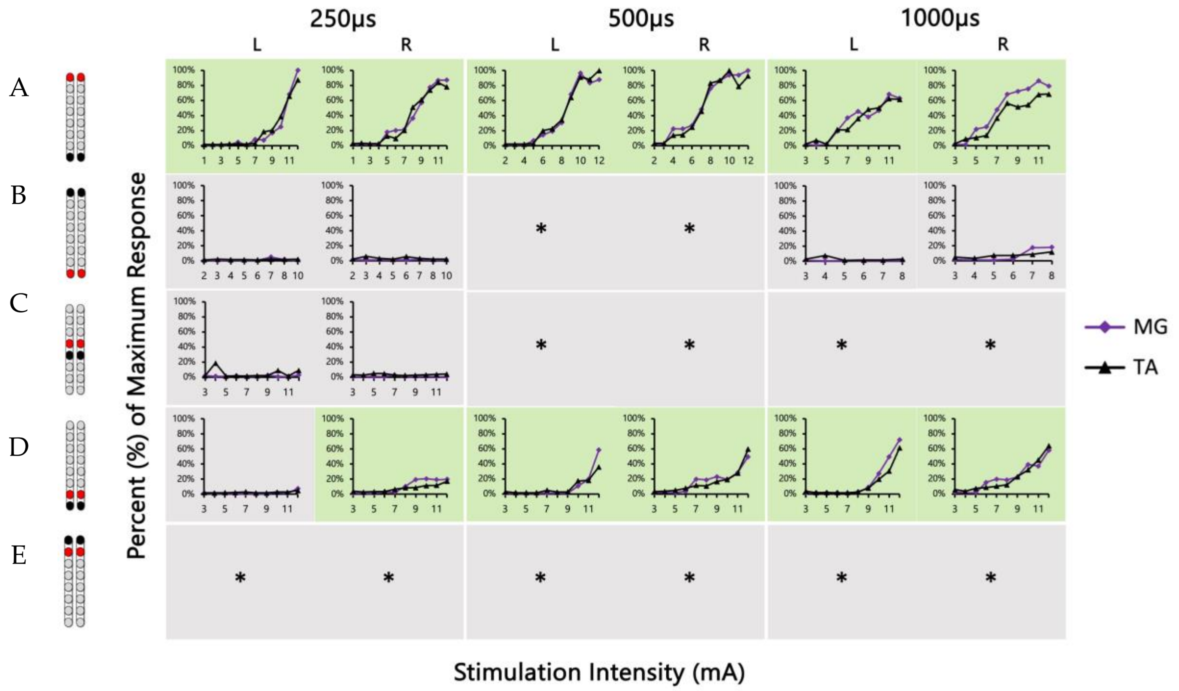

Figure S9. Supine SCES-evoked recruitment curves of the ankle extensor and flexor muscles for 0884. Supine SCES-evoked recruitment curves of the left and right MG and TA muscles collected by stimulating at 2Hz from (1-10mA) using the 5 standardized SCES configurations each collected at pulse durations of 250, 500, and 1000µs. (A) Wide-field configuration with caudal cathodes and rostral anodes. (B) Wide-field configuration with rostral cathodes and caudal anodes. (C) Central narrow-field configuration with rostral anodes and caudal cathodes. (D) Caudal narrow-field configuration with rostral anodes and caudal cathodes. (E) Rostral narrow-field configuration with rostral cathodes and caudal anodes. Recruitment curves highlighted green indicate an overall higher extensor to flexor muscle response, while curves highlighted gray represent an overall higher flexor to extensor muscle response. Responses were normalized to the maximum response of each muscle across all amplitudes in all configurations. Cathode and anode electrodes are shown in black and red, respectively. MG: medial gastrocnemius; TA: tibialis anterior; mA: milliampere; µs: microsecond; L: left; R: right. *Indicates not enough EMG data was available to construct SCES-evoked recruitment curve and/or stimulation resulted in increased abdominal activity.

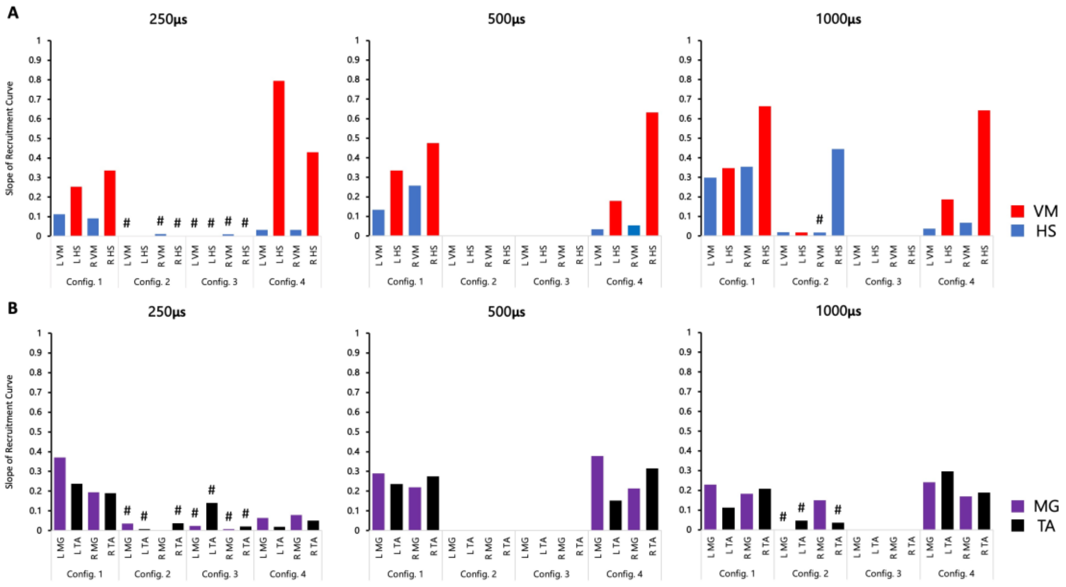

Figure S10. Individual peak slope values of key extensor and flexor muscle pairs for 0884. Maximal peak slope of the SCES-evoked recruitment curves was determined for the knee, hip, and ankle extensors and flexors using the five standardized configurations. (A) Peak slope for the VM and HS at 250, 500, and 1000µs. (B) Peak slope for the GM & RF at 250, 500, and 1000µs. (C) Peak slope for the GM & RF at 250, 500, and 1000µs. Config. 1: wide-field configuration with caudal cathodes and rostral anodes; Config. 2: wide-field configuration with rostral cathodes and caudal anodes; Config. 3: central narrow-field configuration with rostral anodes and caudal cathodes; Config. 4: caudal narrow-field configuration with rostral anodes and caudal cathodes; Config. 5: rostral narrow-field configuration with rostral cathodes and caudal anodes. VM: vastus medialis; HS: hamstrings; MG: medial gastrocnemius; TA: tibialis anterior. # Indicates sigmoidal fit had an R2 < 0.8.

Supplemental Tables [S1-S4]

Table S1.

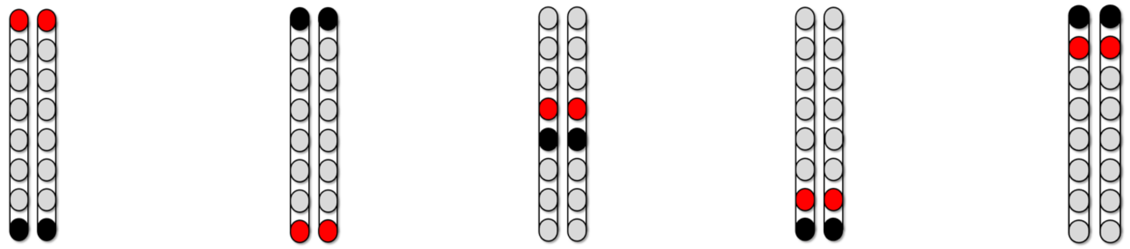

Pulse Width	Configuration 1		Configuration 2		Configuration 3		Configuration 4		Configuration 5	
**250us**	L	R	L	R	L	R	L	R	L	R
**VM**	0.02	0.06	0.39	0.33	0.02	0.01	0.06	0.00	0.00	0.07
**R^2 Fit**	1.00	0.99	1.00	1.00	0.45	0.97	0.86	0.93	0.96	0.98
**HS**	0.40	0.11	0.22	0.22	0.14	0.04	0.45	0.01	0.02	0.26
**R^2 Fit**	0.87	1.00	0.93	0.99	0.79	0.98	0.30	0.92	0.94	0.73
**VM/HS Ratio**	**0.06**	**0.54**	**1.80**	**1.49**	**0.13**	**0.13**	**0.14**	**0.22**	**0.18**	**0.29**
**GM**	0.34	0.18	0.15	0.35	0.10	0.08	0.12	0.00	0.00	0.07
**R^2 Fit**	1.00	0.99	0.96	1.00	0.83	0.94	0.91	0.91	0.92	0.90
**RF**	0.15	0.01	0.34	0.24	0.04	0.01	0.08	0.00	0.00	0.06
**R^2 Fit**	1.00	0.99	1.00	1.00	0.91	0.92	0.93	0.69	0.93	0.97
**GM/RF Ratio**	**2.31**	**12.74**	**0.43**	**1.46**	**2.51**	**12.69**	**1.50**	**2.57**	**0.76**	**1.08**
**MG**	0.21	0.19	0.08	0.02	0.07	0.03	0.11	0.03	0.03	0.04
**R^2 Fit**	0.97	0.99	0.98	0.98	0.86	0.87	0.58	1.00	0.99	0.98
**TA**	0.05	0.18	0.10	0.19	0.02	0.07	0.01	0.39	0.04	0.05
**R^2 Fit**	0.99	0.99	0.98	0.98	0.90	0.86	0.95	0.23	0.96	1.00
**MG/TA Ratio**	**4.08**	**1.09**	**0.76**	**0.10**	**3.73**	**0.49**	**9.64**	**0.07**	**0.73**	**0.84**
**500us**										
**VM**	0.12	0.11	0.36	0.38	0.04	0.05	0.12	0.01	0.42	0.18
**R^2 Fit**	0.82	0.99	1.00	1.00	0.96	0.95	0.91	0.90	1.00	0.99
**HS**	0.26	0.15	0.26	0.31	0.07	0.04	0.29	0.11	0.12	0.32
**R^2 Fit**	0.96	0.99	0.91	1.00	0.91	0.99	0.30	0.96	0.99	0.88
**VM/HS Ratio**	**0.47**	**0.72**	**1.39**	**1.23**	**0.62**	**1.34**	**0.41**	**0.11**	**3.50**	**0.55**
**GM**	0.26	0.39	0.17	0.41	0.09	0.07	0.14	0.04	0.12	0.12
**R^2 Fit**	0.98	0.98	0.99	0.88	0.87	0.98	0.59	0.99	0.99	0.96
**RF**	0.16	0.10	0.35	0.34	0.06	0.04	0.16	0.00	0.38	0.14
**R^2 Fit**	1.00	0.98	1.00	1.00	0.99	1.00	0.74	0.83	1.00	0.99
**GM/RF Ratio**	**1.62**	**3.97**	**0.49**	**1.21**	**1.62**	**1.81**	**0.90**	**12.54**	**0.32**	**0.84**
**MG**	0.23	0.50	0.14	0.08	0.05	0.03	0.15	0.08	0.06	0.06
**R^2 Fit**	0.94	0.96	0.94	0.88	0.99	0.98	0.58	0.74	0.99	0.86
**TA**	0.16	0.33	0.12	0.29	0.02	0.06	0.05	0.44	0.07	0.15
**R^2 Fit**	0.97	0.95	0.99	0.98	0.99	0.98	0.76	0.17	0.99	1.00
**MG/TA Ratio**	**1.39**	**1.51**	**1.19**	**0.26**	**1.95**	**0.53**	**2.93**	**0.17**	**0.83**	**0.37**
**1000us**										
**VM**	0.14	0.14	0.34	0.35	0.05	0.04	0.09	0.03	0.24	0.21
**R^2 Fit**	0.97	0.97	1.00	1.00	0.94	0.93	0.98	0.92	0.99	0.95
**HS**	0.53	0.33	0.39	0.33	0.35	0.08	0.22	0.08	0.27	0.53
**R^2 Fit**	0.97	0.99	0.92	1.00	0.31	0.80	0.44	1.00	1.00	0.97
**VM/HS Ratio**	**0.26**	**0.41**	**0.86**	**1.07**	**0.13**	**0.54**	**0.39**	**0.39**	**0.87**	**0.40**
**GM**	0.29	0.25	0.20	0.38	0.43	0.09	0.22	0.10	0.05	0.51
**R^2 Fit**	0.94	0.76	0.86	0.92	0.21	0.70	0.79	0.95	0.99	0.88
**RF**	0.16	0.08	0.31	0.29	0.09	0.03	0.18	0.02	0.18	0.27
**R^2 Fit**	0.94	0.99	1.00	1.00	0.36	0.95	0.98	0.97	0.98	0.98
**GM/RF Ratio**	**1.82**	**2.92**	**0.64**	**1.28**	**4.93**	**2.80**	**1.22**	**4.85**	**0.25**	**1.89**
**MG**	0.20	0.19	0.36	0.11	0.10	0.05	0.41	0.08	0.24	0.07

Table S1. (Continued…)

**R^2 Fit**	0.93	0.97	0.97	0.99	0.89	0.65	0.81	0.94	0.99	0.78
**TA**	0.45	0.41	0.13	0.32	0.04	0.11	0.04	0.46	0.22	0.19
**R^2 Fit**	0.90	0.81	0.98	0.98	0.65	0.43	0.96	0.49	0.75	1.00
**MG/TA Ratio**	**0.46**	**0.46**	**2.71**	**0.35**	**2.84**	**0.44**	**9.34**	**0.18**	**1.09**	**0.37**

Table S2.

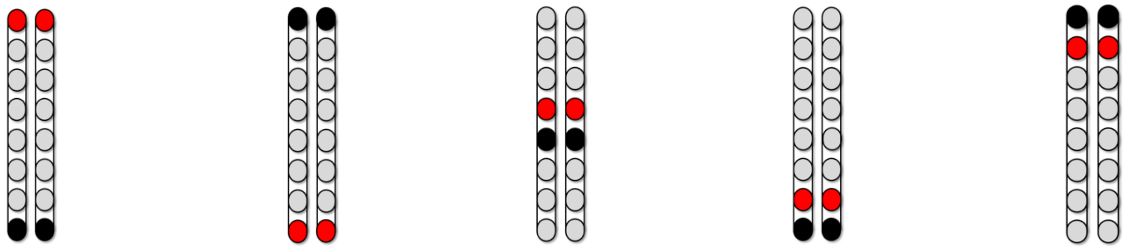

Pulse Width	Configuration 1		Configuration 2		Configuration 3		Configuration 4		Configuration 5	
**250us**	L	R	L	R	L	R	L	R	L	R
**VM**	0.11	0.10	0.11	0.09	0.43	0.14	0.06	0.08	0.01	0.03
**R^2 Fit**	0.97	0.93	1.00	0.99	0.73	0.78	0.98	1.00	0.40	1.00
**HS**	0.30	0.59	0.25	0.39	0.82	0.81	0.24	0.35	0.01	0.01
**R^2 Fit**	0.98	0.99	1.00	1.00	0.86	0.99	0.98	0.99	0.53	1.00
**VM/HS Ratio**	**0.38**	**0.17**	**0.46**	**0.24**	**0.52**	**0.17**	**0.26**	**0.24**	**0.90**	**1.91**
**GM**	0.42	0.66	0.11	0.34	0.79	0.55	0.37	0.31	0.01	0.02
**R^2 Fit**	0.97	0.80	0.93	0.76	0.50	0.36	1.00	0.98	1.00	0.88
**RF**	0.26	0.37	0.07	0.18	0.82	0.34	0.04	0.38	0.01	0.02
**R^2 Fit**	0.99	0.99	0.99	0.93	0.96	0.88	0.97	1.00	0.70	1.00
**GM/RF Ratio**	**1.59**	**1.76**	**1.55**	**1.86**	**0.96**	**1.63**	**8.75**	**0.81**	**0.68**	**0.67**
**MG**	0.39	0.43	0.28	0.14	0.33	0.18	0.38	0.36	0.15	0.08
**R^2 Fit**	0.90	0.92	1.00	0.99	0.67	0.95	0.99	1.00	0.54	0.43
**TA**	0.37	0.33	0.16	0.10	0.32	0.37	0.33	0.19	0.03	0.01
**R^2 Fit**	0.87	0.95	1.00	0.99	0.84	0.93	0.98	1.00	0.51	0.30
**MG/TA Ratio**	**1.05**	**1.30**	**1.77**	**1.40**	**1.02**	**0.48**	**1.15**	**1.89**	**4.55**	**9.04**
**500us**										
**VM**	0.60	0.48	0.11	0.07	0.47	0.49	0.11	0.14	0.09	0.15
**R^2 Fit**	1.00	0.95	1.00	0.92	1.00	0.95	0.98	0.81	1.00	1.00
**HS**	0.52	0.62	0.28	0.37	0.36	0.64	0.28	0.60	0.18	0.29
**R^2 Fit**	1.00	0.99	1.00	0.98	0.99	0.99	1.00	1.00	1.00	1.00
**VM/HS Ratio**	**1.14**	**0.77**	**0.39**	**0.19**	**1.31**	**0.76**	**0.38**	**0.23**	**0.48**	**0.52**
**GM**	0.52	0.28	0.23	0.67	0.49	0.39	0.36	0.63	0.04	0.02
**R^2 Fit**	0.75	0.56	0.79	0.50	0.72	0.96	0.98	0.99	0.89	0.99
**RF**	0.49	0.28	0.07	0.17	0.42	0.34	0.15	0.26	0.08	0.09
**R^2 Fit**	0.97	0.77	1.00	0.60	0.97	0.77	0.95	0.96	0.99	0.99
**GM/RF Ratio**	**1.05**	**1.01**	**3.44**	**3.90**	**1.17**	**1.13**	**2.32**	**2.43**	**0.42**	**0.20**
**MG**	0.17	0.20	0.10	0.16	0.18	0.21	0.32	0.31	0.01	0.09
**R^2 Fit**	1.00	0.97	0.97	0.80	1.00	0.97	0.96	0.97	1.00	1.00
**TA**	0.18	0.23	0.05	0.14	0.19	0.23	0.34	0.30	0.03	0.05
**R^2 Fit**	0.99	0.97	0.98	0.88	0.99	0.96	0.97	0.98	1.00	0.96
**MG/TA Ratio**	**0.94**	**0.89**	**1.76**	**1.16**	**0.91**	**0.93**	**0.93**	**1.02**	**0.42**	**1.83**
**1000us**										
**VM**	0.16	0.37	0.13	0.11	0.41	0.20	0.13	0.25	0.15	0.22
**R^2 Fit**	0.97	0.68	0.97	0.91	0.98	0.98	0.99	0.82	0.98	0.95
**HS**	0.49	0.52	0.41	0.66	0.50	0.58	0.33	0.82	0.17	0.28
**R^2 Fit**	0.96	0.98	0.97	1.00	0.97	1.00	0.97	1.00	0.99	0.97
**VM/HS Ratio**	**0.33**	**0.72**	**0.32**	**0.16**	**0.83**	**0.34**	**0.39**	**0.30**	**0.90**	**0.79**
**GM**	0.50	0.93	0.64	0.86	0.58	0.37	0.30	0.69	0.13	0.03
**R^2 Fit**	0.85	0.37	0.39	0.48	0.54	0.85	1.00	0.99	0.99	0.98
**RF**	0.30	0.48	0.07	0.38	0.49	0.35	0.20	0.58	0.06	0.08
**R^2 Fit**	0.94	0.60	0.99	0.31	0.98	0.91	0.99	0.82	0.98	0.98
**GM/RF Ratio**	**1.67**	**1.91**	**8.64**	**2.24**	**1.19**	**1.08**	**1.48**	**1.18**	**2.12**	**0.43**
**MG**	0.69	0.66	0.63	0.74	0.25	0.18	0.62	0.33	0.00	0.02

Table S2. (Continued…)

**R^2 Fit**	0.92	0.58	0.59	0.48	1.00	1.00	0.99	0.99	0.66	0.19
**TA**	0.63	0.58	0.61	0.59	0.35	0.21	0.59	0.31	0.08	0.02
**R^2 Fit**	0.93	0.84	0.49	0.33	1.00	0.99	0.98	0.96	0.16	0.96
**MG/TA Ratio**	**1.11**	**1.15**	**1.04**	**1.25**	**0.71**	**0.86**	**1.06**	**1.06**	**0.02**	**0.88**

Table S3.

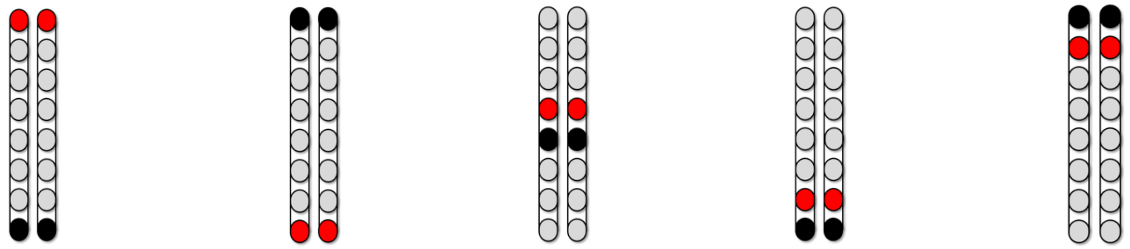

Pulse Width	Configuration 1		Configuration 2		Configuration 3		Configuration 4		Configuration 5	
**250us**	L	R	L	R	L	R	L	R	L	R
**VM**	0.06	0.15	0.10	0.00	0.00	0.00	0.00	0.18	0.01	0.00
**R^2 Fit**	0.13	0.78	0.69	0.95	0.37	0.40	0.51	1.00	0.07	0.85
**HS**	0.02	0.28	0.01	0.02	0.01	0.00	0.01	0.03	0.00	0.00
**R^2 Fit**	0.78	1.00	0.87	0.94	0.33	0.90	0.56	0.98	0.99	0.72
**VM/HS Ratio**	**3.95**	**0.55**	**9.24**	**0.13**	**0.35**	**1.05**	**0.14**	**5.12**	**23.62**	**0.44**
**GM**	*	*	*	*	*	*	*	*	*	*
**R^2 Fit**	*	*	*	*	*	*	*	*	*	*
**RF**	0.01	0.16	0.06	0.03	0.00	0.02	0.00	0.14	0.00	0.01
**R^2 Fit**	0.28	0.98	0.72	0.99	0.44	0.77	0.99	0.96	0.99	0.33
**GM/RF Ratio**	*	*	*	*	*	*	*	*	*	*
**MG**	0.00	0.16	0.00	0.02	0.01	0.01	0.00	0.10	0.00	0.01
**R^2 Fit**	0.84	0.96	0.95	0.80	0.20	0.61	0.76	0.96	0.76	0.39
**TA**	0.05	0.09	0.04	0.03	0.02	0.02	0.06	0.06	0.01	0.03
**R^2 Fit**	0.38	0.94	0.24	0.26	0.77	0.50	0.73	0.97	0.58	0.12
**MG/TA Ratio**	**0.10**	**1.73**	**0.03**	**0.85**	**0.30**	**0.67**	**0.08**	**1.64**	**0.14**	**0.25**
**500us**										
**VM**	0.22	0.16	0.55	0.04	0.03	0.02	0.00	0.16	0.34	0.01
**R^2 Fit**	1.00	0.97	1.00	1.00	0.99	0.81	0.70	0.99	1.00	1.00
**HS**	0.12	0.22	0.59	0.03	0.02	0.02	0.01	0.16	0.30	0.02
**R^2 Fit**	1.00	1.00	1.00	0.99	0.72	0.95	0.94	0.93	1.00	1.00
**VM/HS Ratio**	**1.82**	**0.71**	**0.94**	**1.53**	**1.69**	**0.68**	**0.50**	**0.97**	**1.14**	**0.64**
**GM**	*	*	*	*	*	*	*	*	*	*
**R^2 Fit**	*	*	*	*	*	*	*	*	*	*
**RF**	0.18	0.14	0.54	0.07	0.02	0.21	0.00	0.10	0.27	0.03
**R^2 Fit**	1.00	0.93	1.00	0.98	0.99	0.81	0.72	1.00	1.00	0.98
**GM/RF Ratio**	*	*	*	*	*	*	*	*	*	*
**MG**	0.44	0.18	0.04	0.09	0.01	0.01	0.00	0.26	0.01	0.02
**R^2 Fit**	0.74	0.98	1.00	0.64	0.62	0.47	0.67	0.58	0.94	0.96
**TA**	0.03	0.06	0.13	0.03	0.05	0.02	0.05	0.10	0.05	0.02
**R^2 Fit**	0.64	0.99	0.68	0.92	0.29	0.87	0.38	0.95	0.58	0.62
**MG/TA Ratio**	**17.50**	**2.85**	**0.33**	**3.43**	**0.25**	**0.53**	**0.01**	**2.61**	**0.12**	**0.76**
**1000us**										
**VM**	0.39	0.12	0.31	0.16	0.10	0.02	0.03	0.28	0.05	0.00
**R^2 Fit**	0.83	1.00	1.00	0.53	0.99	0.85	0.18	0.93	1.00	0.46
**HS**	0.50	0.27	0.37	0.20	0.01	0.03	0.01	0.33	0.08	0.01
**R^2 Fit**	0.80	1.00	1.00	0.93	0.92	0.96	0.55	0.98	0.99	1.00
**VM/HS Ratio**	**0.79**	**0.44**	**0.82**	**0.81**	**6.95**	**0.66**	**2.81**	**0.83**	**0.67**	**0.12**
**GM**	*	*	*	*	*	*	*	*	*	*
**R^2 Fit**	*	*	*	*	*	*	*	*	*	*
**RF**	0.38	0.22	0.43	0.19	0.02	0.06	0.00	0.19	0.06	0.01
**R^2 Fit**	0.83	1.00	0.99	0.74	0.83	0.74	0.55	0.91	1.00	0.33
**GM/RF Ratio**	*	*	*	*	*	*	*	*	*	*
**MG**	0.49	0.10	0.18	0.06	0.00	0.02	0.02	0.29	0.00	0.00

Table S3. (Continued…)

**R^2 Fit**	0.95	0.95	0.99	0.69	0.87	0.87	0.96	0.87	0.64	0.00
**TA**	0.11	0.05	0.22	0.08	0.05	0.04	0.03	0.14	0.05	0.01
**R^2 Fit**	0.68	0.98	0.83	0.33	0.77	0.53	0.20	0.95	0.20	0.35
**MG/TA Ratio**	**4.47**	**1.86**	**0.80**	**0.75**	**0.08**	**0.59**	**0.58**	**2.06**	**0.04**	**0.38**

Table S4.

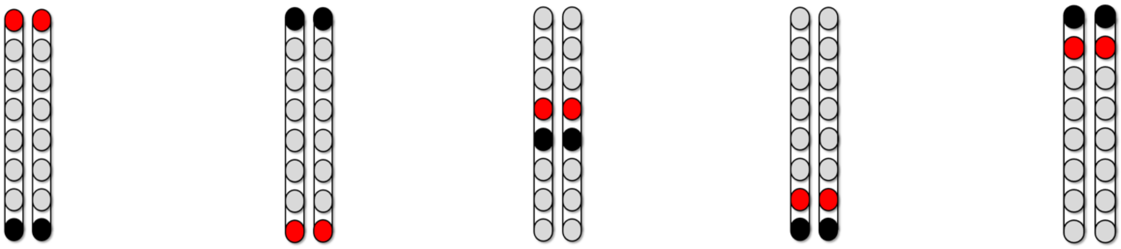

Pulse Width	Configuration 1		Configuration 2		Configuration 3		Configuration 4		Configuration 5	
**250us**	L	R	L	R	L	R	L	R	L	R
**VM**	0.11	0.09	0.00	0.01	0.00	0.01	0.03	0.03	*	*
**R^2 Fit**	1.00	0.98	0.73	0.83	0.12	0.35	0.98	0.99	*	*
**HS**	0.25	0.34	0.00	0.00	0.00	0.00	0.79	0.43	*	*
**R^2 Fit**	1.00	0.94	0.80	0.01	0.93	0.20	0.77	0.98	*	*
**VM/HS Ratio**	**0.45**	**0.27**	**0.56**	**8.11**	**2.57**	**4.68**	**0.04**	**0.08**	*	*
**GM**	*	*	*	*	*	*	*	*	*	*
**R^2 Fit**	*	*	*	*	*	*	*	*	*	*
**RF**	0.19	0.19	0.01	0.01	0.00	0.01	0.05	0.04	*	*
**R^2 Fit**	1.00	1.00	0.98	0.96	0.63	0.05	0.98	0.99	*	*
**GM/RF Ratio**	*	*	*	*	*	*	*	*	*	*
**MG**	0.37	0.19	0.04	0.00	0.02	0.01	0.06	0.08	*	*
**R^2 Fit**	0.99	0.98	0.39	0.08	0.07	0.11	0.71	0.99	*	*
**TA**	0.24	0.19	0.01	0.04	0.14	0.02	0.02	0.05	*	*
**R^2 Fit**	0.99	0.99	0.43	0.24	0.26	0.19	0.74	0.96	*	*
**MG/TA Ratio**	**1.55**	**1.03**	**4.77**	**0.08**	**0.17**	**0.33**	**3.24**	**1.55**	*	*
**500us**										
**VM**	0.13	0.26	*	*	*	*	0.03	0.05	*	*
**R^2 Fit**	1.00	0.96	*	*	*	*	1.00	0.99	*	*
**HS**	0.33	0.47	*	*	*	*	0.18	0.63	*	*
**R^2 Fit**	1.00	0.99	*	*	*	*	0.98	0.98	*	*
**VM/HS Ratio**	**0.40**	**0.54**	*	*	*	*	**0.19**	**0.09**	*	*
**GM**	*	*	*	*	*	*	*	*	*	*
**R^2 Fit**	*	*	*	*	*	*	*	*	*	*
**RF**	0.18	0.25	*	*	*	*	0.11	0.06	*	*
**R^2 Fit**	1.00	1.00	*	*	*	*	1.00	1.00	*	*
**GM/RF Ratio**	*	*	*	*	*	*	*	*	*	*
**MG**	0.29	0.22	*	*	*	*	0.38	0.21	*	*
**R^2 Fit**	0.98	0.98	*	*	*	*	0.94	0.88	*	*
**TA**	0.24	0.27	*	*	*	*	0.15	0.32	*	*
**R^2 Fit**	0.98	0.97	*	*	*	*	0.95	0.93	*	*
**MG/TA Ratio**	**1.23**	**0.80**	*	*	*	*	**2.47**	**0.68**	*	*
**1000us**										
**VM**	0.30	0.35	0.02	0.02	*	*	0.04	0.07	*	*
**R^2 Fit**	0.97	0.98	0.95	0.71	*	*	0.99	0.99	*	*
**HS**	0.35	0.66	0.02	0.44	*	*	0.19	0.64	*	*
**R^2 Fit**	0.98	0.98	0.99	0.98	*	*	1.00	0.94	*	*
**VM/HS Ratio**	**0.86**	**0.53**	**1.11**	**0.04**	*	*	**0.20**	**0.11**	*	*
**GM**	*	*	*	*	*	*	*	*	*	*
**R^2 Fit**	*	*	*	*	*	*	*	*	*	*
**RF**	0.21	0.23	0.03	0.03	*	*	0.10	0.08	*	*
**R^2 Fit**	0.99	1.00	1.00	0.98	*	*	1.00	1.00	*	*
**GM/RF Ratio**	*	*	*	*	*	*	*	*	*	*
**MG**	0.23	0.18	0.00	0.15	*	*	0.24	0.17	*	*

Table S4. (Continued…)

**R^2 Fit**	0.93	0.98	0.96	1.00	*	*	1.00	0.94	*	*
**TA**	0.11	0.21	0.05	0.04	*	*	0.30	0.19	*	*
**R^2 Fit**	0.98	0.96	0.57	0.90	*	*	0.99	1.00	*	*
**MG/TA Ratio**	**2.03**	**0.88**	**0.02**	**4.06**	*	*	**0.81**	**0.90**	*	*

Supplementary Methods

Process for obtaining recruitment curve slopes:

1) Recruitment data is obtained during mapping with EMGs. Values are normalized to the maximum evoked potential for each muscle.

2) Matlab code is used to create recruitment curves and fit them to an optimized sigmoid shape.

3) Somerecruitment curves were abnormal and therefore did not fit well to an optimized sigmoid. These curves were excluded/included by visual inspection. A cutoff value of R^2 = 0.8 was deemed a strong effect size, but this could be overridden by visual inspection.

4) If a curve was chosen to be included, a 6th order polynomial fit was applied using Matlab. The maximum slope of this fit is the slope of the recruitment curve. Applying Sigmoid Fits: Applying 6th Order Polynomial Fits.

Applying Sigmoid Fits:

Updated Methodology:

To create optimal initial guesses to pass to **lsqcurvefit** for each recruitment curve, a particle swarm optimization was performed to find the initial guess parameters that generated the greatest R^2 value. This optimization works by creating an equidistant spread of 'particles' in a 4-dimensional space (4 parameters), and assessing the dimensions of each particle to find the one that creates the greatest R^2:

```
options = optimoptions('particleswarm', 'Display', 'off', 'SwarmSize', 500, ...
'MaxIterations', 500);

bestInitialGuesses_PSO = particleswarm(@(params) -objectiveFunction(params,...
amplitudes, recruitment), 4, [0 0 0 0], [1 1 10 5], options);

  function rSquared = objectiveFunction(params, amplitudes, recruitment)
           sigFit = sigmoid(params, amplitudes);
           recMean = mean(recruitment);
           totalSumSquares = sum((recruitment - recMean).^2);
           residualSumSqaures = sum((recruitment - sigFit).^2);
           rSquared = 1 - (residualSumSqaures/totalSumSquares);
  end

params_fit = lsqcurvefit(sigmoid,bestInitialGuesses_PSO,amplitudes,recruitment);
sigmoidFunction = sigmoid(params_fit, amplitudes);
```

The size of the swarm, maximum iterations, and bounds of parameters can be adjusted to create more accurate optimizations. For our case, the following options were determined suitable:

- Swarm size = 500
- Maximum iterations = 500
- Lower bounds = 0,0,0,0
- Upper bounds = 1,1,10,5

Applying 6th Order Polynomial Fits:

```
coefficients = polyfit(amplitudesToPlot, sensorSpecificData, 6);
yfit = polyval(coefficients, amplitudesToPlot);
% display ploynomial fit for processing:
disp("Polynomial fit:")
disp(yfit);
plot(app.Method3Axes, amplitudesToPlot, yfit, "Color","g","LineWidth",3);
```
